# F106. STATE AND TRAIT RELATED NATURE OF INSIGHT IMPAIRMENT IN SCHIZOPHRENIA

**DOI:** 10.1093/schbul/sby017.637

**Published:** 2018-04-01

**Authors:** Lebogang Phahladira, Bonginkosi Chiliza, Laila Asmal, Sanja Kilian, Freda Scheffler, Robin Emsley

**Affiliations:** 1 Stellenbosch University; 2 University of KwaZulu Natal

## Abstract

**Background:**

Impairment of insight is a prominent feature of schizophrenia and is associated with poor adherence and poor outcomes. While many studies have investigated the nature of insight impairment in schizophrenia, few have charted its course longitudinally. In this study we investigated changes in different components of insight during the first 12 months of antipsychotic treatment.

**Methods:**

The sample comprised 107 never or minimally treated patients with a first episode of schizophrenia, schizophreiform or schizoaffective disorder. They were treated according to a fixed protocol with flupenthixol decanoate. Insight was assessed with the self-rating Birchwood Insight Scale and the investigator rated global insight item of the PANSS scale.Psychopathology was assessed with the PANSS and CDSS. Cognitive performance was assessed with the MATRICS. We performed evaluations at baseline, month 6 and month 12. Linear mixed effects mixed models for repeated measures were conducted to assess changes over time, adjusting for age, gender and educational status.

**Results:**

There were no significant changes in the BIS subscales of symptom awareness, awareness of illness or total BIS score. The need for treatment subscale improved slightly (p=0.02) while the PANSS global insight improved considerably (p<0.0001). Degree of insight impairment was only weakly correlated with psychopathology and cognition

**Discussion:**

Insight impairment is common in schizophrenia and displays trait-like rather than state-like features. These findings have important clinical implications.

